# Impact of advice to quit smoking on rating of care and anticipation of stigma in primary care

**DOI:** 10.1017/S1463423625100273

**Published:** 2025-07-11

**Authors:** Chris Barton, Max Wouterlood, Melis Selamoglu, Sanduni Madawala, Joanne Enticott, Elizabeth Sturgiss, Johnson George, Ron Borland

**Affiliations:** 1 School of Public Health and Preventive Medicine, Monash University, Melbourne, Australia; 2 Monash Centre for Health Research and Implementation, School of Public Health and Preventive Medicine, Monash University, Clayton, VIC 3168, Australia; 3 School of Primary and Allied Health Care, Monash University, Melbourne, Australia; 4 Centre for Medicine Use and Safety and School of Public Health and Preventive Medicine, Monash University, Melbourne, Australia; 5 School of Psychology, Deakin University, Melbourne, Australia

**Keywords:** General practice, patient assessment/satisfaction, patient experience, smoking cessation

## Abstract

**Aim:**

We assessed patient experience of care, comparing current and past smokers, and whether frequency of advice to quit smoking impacts patient rating of care.

**Background:**

Experience of care may be a concern for people who smoke and affect their partnership with healthcare providers.

**Methods:**

We surveyed adults aged over 35 years who had visited a general practitioner (GP) for health care in the past year (n = 611 current and n = 275 ex-smokers). Questions assessed smoking history, experience of care, anticipation of stigma, and perceptions of smoking cessation advice received in general practice.

**Findings:**

Fewer than half (48.8%) of current smokers reported ‘always’ or ‘usually’ being advised to quit smoking, or being advised in a way that motivated them to quit by a GP or other care provider at their GP practice. Current smokers tended to delay or avoid help seeking when needed and experienced more anticipation of stigma in healthcare settings. Conversely, respondents who reported being advised to quit more frequently rated overall quality of care more highly. These data show that asking about smoking and providing advice to quit smoking was acceptable to most respondents and associated with higher ratings of quality of care. However, advice should be provided in a way that motivates patients, without exacerbating the stigma associated with smoking, which may impact help seeking.

## Introduction

Family doctors play an important role in motivating patients who smoke to make the decision to stop smoking and to provide relevant treatment, referral, and/or support to do so (Manolios *et al.*, 2021, Borland *et al.*, 2008, Burrows and Carlisle, 2010). Brief advice to patients from their family physician or general practitioner (GP) to stop smoking is effective (Lancaster and Stead, 2004, Zwar and Richmond, 2006), however, doctors identify smokers and deliver advice to quit smoking at only modest rates (Papadakis *et al.*, 2010, Bryant *et al.*, 2015). Barriers to providing smoking cessation advice include GPs’ beliefs that intervention will not change the patient’s smoking behaviour, competing clinical priorities, and insufficient time, as well as a lack of knowledge and training (Nelson *et al.*, 2015, Vogt *et al.*, 2005). GPs’ perceptions of patient engagement and patients’ responses to advice about smoking cessation can influence their advice giving (Coleman *et al.*, 2000, Cunningham, 2014) as well as a desire to maintain a good relationship with patients (Coleman *et al.*, 2000, Coleman *et al.*, 2004, Cunningham, 2014, Codern-Bove *et al.*, 2014)

Where smokers report positive experiences with health professionals, they show a willingness to further engage with health providers to help with smoking cessation (Vuong *et al.*, 2016). Active referral of smokers to Quitline services appears acceptable to both GPs and their patients (Borland *et al.*, 2008). On the other hand, concerns persist that patients may express anger at being told what to do by health professionals and resent actions towards attempts at external control by health providers (Burrows and Carlisle, 2010). Highly directive and recurrent, or unwelcome smoking cessation advice, can be counterproductive (Burrows and Carlisle, 2010, Hansen and Nelson, 2011, Irvine *et al.*, 1999). Interactions with health professionals can serve to reinforce feelings of being judged and blamed for past smoking behaviours, and smokers may be particularly vulnerable to poor patient experience of care and stigma in health settings (Boland *et al.*, 2017, Madawala *et al.*, 2023c).

Qualitative studies suggest that patient experience is an area of concern for patients who smoke, whose anticipation of stigma in healthcare settings can affect the disclosure of information and effective partnership with healthcare providers, particularly in the context of smoking-related chronic illness or cancer (Scott *et al.*, 2015, Carter-Harris, 2015, Oliver, 2001, Madawala *et al.*, 2023b). Smokers internalizing smoking-related negative stereotypes has been associated with increased resistance to smoking cessation advice and the purposeful non-disclosure of smoking status to healthcare providers (Evans-Polce *et al.*, 2015, Halladay *et al.*, 2015). Smokers experience stigma in a variety of contexts and these feelings are particularly harmful within healthcare settings where ‘anticipated’ stigma may act as a barrier to accessing health care in the future or delaying seeking care until a crisis point (Earnshaw *et al.*, 2012, Earnshaw and Quinn, 2011).

About 10% of smokers in Australia will speak with a GP for help to quit smoking (Australian Institute of Health and Welfare, 2020). Smokers expect to be asked about smoking by their GP and consider it to be both part of their GPs clinical duties as well as showing concern for their health (Papadakis *et al.*, 2020, Manolios *et al.*, 2021). Large studies of smokers’ experiences of care and ratings of health providers in the USA found that among patients enrolled in Medicaid and Medicare programmes, the frequency of discussions about smoking cessation was positively associated with the overall rating of healthcare providers (Holla *et al.*, 2018, Winpenny *et al.*, 2017). Healthcare providers who ‘always’ asked about smoking were rated more highly than those who did not (Winpenny *et al.*, 2017). The rating of Medicaid patients’ physicians increased as they received at least some smoking cessation advice. The rating increased even more when options for smoking cessation medications or other smoking cessation methods were discussed. Both studies concluded that giving smoking cessation advice is not detrimental to the experience of care and may reflect better quality of care among Medicaid and Medicare patient populations.

This study aimed to determine if differences exist in the perceived experience of primary care between a general population of primary care patients who currently smoke or had recently quit smoking and if the frequency of advice to quit smoking from GPs or other healthcare providers at a GP’s practice is associated with ratings of health care of Australian general practitioners.

## Methods

### Participants and sampling

A cross-sectional, nationwide survey of Australian smokers and ex-smokers was undertaken between December 2019 and January 2020, prior to the introduction of COVID-19 restrictions in Australia. A link to the study survey was promoted on social media using paid adverts on Facebook and Instagram. Two rounds of trial ads were tested using six different combinations of images and text before deciding on three final ads that were run until the estimated minimum sample size (n = 180 per group (smokers/past smokers) had been exceeded. Individuals who clicked on the study advertisement were taken to an online survey in Qualtrics XM software (Qualtrics, Seattle, WA). Respondents completed a set of screening questions to assess eligibility which included age (≥ 35 years old), smoking status (current smoker or ex-smoker who had quit within the last 5 years), living in Australia, and having visited a GP for their own health in the last 12 months. Respondents were offered the opportunity to enter into a prize draw to win one of 12, $25 gift vouchers.

The survey landing page contained brief information about the study and a link to the detailed study Explanatory Statement. Consent was indicated by clicking through to the survey from the survey landing page. The study was approved by the Monash University Human Research Ethics Committee (Application No. 17126).

### Data collection

Data were collected from a single, online survey (median completion time 9 minutes). Smoking status was determined by a single question that asked, ‘How often do you now smoke cigarettes, pipes, or other tobacco products?’ Current smokers were defined as participants who smoked either daily, at least once a week, or less than weekly. Participants who self-reported they did not smoke at all, at the time of completing the survey, but who reported having been a regular smoker in the past, were defined as ex-smokers. Amongst current smokers, tobacco dependency was assessed using questions from the Fagerstrom nicotine dependence test (Heatherton *et al.*, 1991).

Questions about quit smoking advice from GPs or other healthcare providers at their GP practice were adapted for an Australian setting from Winpenny et al (Winpenny *et al.*, 2017). We asked, ‘In the last 12 months how often, if at all, were you advised to quit smoking by a GP or other healthcare provider at your GP’s practice?’ Respondents who had been advised to quit smoking were subsequently asked, ‘Did they advise you in a way that made you feel motivated to try to quit?’, ‘did you try to quit smoking?’, ‘how important do you think it is for you to quit smoking?’, and ‘when did you last attempt to quit?’ Ex-smokers were asked, ‘Do you think help from a GP contributed to you quitting successfully?’

Patient experience questions were derived from the Australian Bureau of Statistics (ABS) Patient Experience Survey (PEx) (General Practice questions sub-set) (Australian Bureau of Statistics, 2019) and the Consumer Assessment of Health Care Providers and Systems (CAHPS) survey. The ABS PEx survey assesses the experience of health services at a system level. That is, questions are not asked about encounters with individual providers, but with all GPs a patient has seen in the past 12 months. This is particularly appropriate for the Australian healthcare system in which patients are able to choose their primary care provider and do not need to register with a single provider or general practice. They are able to seek and receive care from different healthcare providers within a practice or from different practices.

In addition to the ABS PEx survey questions, questions adapted from the CAHPS survey were used to assess perceptions of the broader clinic environment and overall rating of care. Two questions were asked about patient views on how they were treated by clerks and receptionists in the practice. Overall rating of care was assessed by the question ‘using any number from 0 to 10, where 0 is the worst care possible and 10 is the best care possible, what number would you use to rate the care received from GPs?’

We determined the degree to which respondents ‘anticipated’ experiencing stigma in primary care settings using four items from the Chronic Illness Anticipated Stigma Scale – healthcare provider sub-scale (Earnshaw *et al.*, 2013) (A healthcare worker will – be frustrated with you; will give you poor care; will blame you for not getting better; will think you’re a bad patient). Each item was scored on a five-point Likert scale ranging from very unlikely to very likely. This instrument has been shown previously to have good reliability and validity among people living with chronic illnesses (Earnshaw *et al.*, 2013). Finally, respondents answered questions about self-perceived health status, demographic, and social circumstances. These questions included age, gender, postcode (from which we determined the relative level of socio-economic advantage and disadvantage) (Australian Bureau of Statistics, 2018), highest level of education, availability of social support, employment, and marital status.

### Data analysis

**Figure 1.**
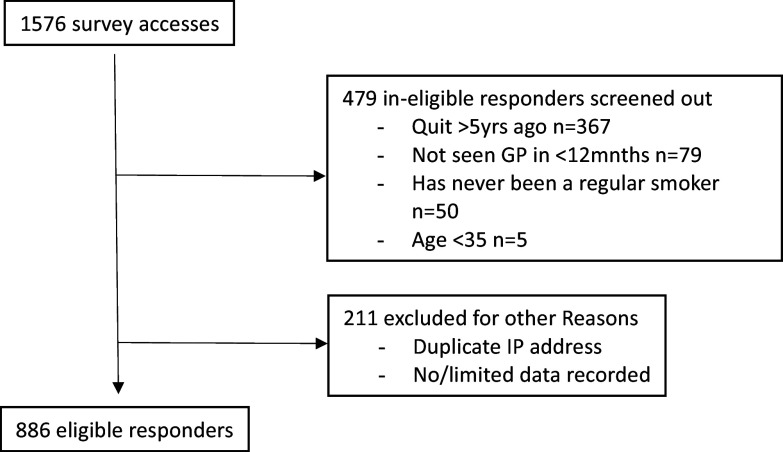
PRISMA flow diagram showing the flow of participants into the study and reasons for exclusion.

Data were downloaded from Qualtrics to SPSS Version 28 for analysis (IBM SPSS Statistics for Windows, Armonk, NY, 2021). We checked the user IP address, and where the same IP address was recorded more than once, we retained data from the first record only for analysis. Respondents who completed the screening questions but did not answer any questions in the main question set were also removed prior to analysis (Figure 1).

Differences in socio-demographic characteristics of smokers and ex-smokers were assessed using Chi-Squared tests for categorical variables and independent sample t-tests or One-Way Analysis of Variance (ANOVA) for continuous variables. Questions concerning the patient experience of primary care were divided into domains that included access to healthcare services, the clinic environment, relational experiences, and overall rating of care from GPs. A small number of current smokers (n = 19, 3.1%) were identified as using ‘only other’ tobacco products and were excluded from subsequent analyses. Logistic regression was used to determine differences between smokers’ and ex-smokers’ experiences of care across each domain of patient experience of care, adjusting for socio-demographic characteristics and self-rated health. Secondly, among current smokers, odds ratios were calculated from logistic regression to determine associations between the binary variables ‘advised to quit smoking’ (sometimes/never vs always/usually), and ‘advised in a way that motivated you to try to quit’ (no/not really vs yes).

P-values less than 0.05 were considered statistically significant.

## Results

A total of 886 responses were retained for analysis (Figure 1). Respondents were either current smokers (n = 611) or ex-smokers who had quit smoking tobacco cigarettes within the last 5 years (n = 275). Ages ranged from 35 to 83 years and respondents reported a mean of 6.9 visits to a GP in the past 12 months (Table 1). Current smokers were more likely to live in metropolitan areas and had significantly poorer self-rated health than ex-smokers (Table 1). Additional characteristics of smokers are presented in Table 2. Most smoked factory-made cigarettes (66.0%) and 11.3% reported dual use of e-cigarettes. More than four in five reported smoking within 30 minutes of waking (Table 2).

**Table 1.** Demographic characteristics of respondents

	Total n = 886	Ex-smoker n = 275 (31%)	Current smoker n = 611 (69%)	P-value
**Sex**				
Male	287 (37.1%)	93 (38.4%)	194 (36.5%)	0.600
Female	487 (62.9%)	149 (61.6%)	338 (63.5%)	
**Age**				
35–44	69 (7.8%)	26 (9.5%)	43 (7.1%)	
45–54	157 (17.8%)	42 (15.3%)	115 (18.9%)	0.156
55–64	370 (41.9%)	108 (39.3%)	262 (43.0%)	
65–74	249 (28.2%)	82 (29.8%)	167 (27.4%)	
75+	39 (4.4%)	17 (6.2%)	22 (3.6%)	
**Education**				
<year 12	327 (41.6%)	103 (42.4%)	224 (41.2%)	
Year 12 or TAFE/diploma	341 (43.3%)	102 (42.0%)	239 (43.9%)	0.874
University degree	119 (15.1%)	38 (15.6%)	38 (15.6%)	
**Marital Status**				
Partnered	391 (50.5%)	133 (55.6%)	258 (48.2%)	
No current partner	375 (48.4%)	103 (43.1%)	272 (50.8%)	0.135
Other	8 (1.0%)	3 (1.3%)	5 (0.9%)	
**State of residence**				
NSW/ACT	175 (19.8%)	50 (18.2%)	125 (20.5%)	
NT	7 (0.8%)	5 (1.8%)	2 (0.3%)	
Qld	187 (21.1%)	58 (21.1%)	129 (21.1%)	
SA	92 (10.4%)	30 (10.9%)	62 (10.1%)	0.301
Tas	39 (4.4%)	15 (5.5%)	24 (3.9%)	
Vic	174 (19.6%)	55 (20.0%)	119 (19.5%)	
WA	94 (10.6%)	24 (8.7%))	70 (11.5%	
**Remoteness**				
Major city	395 (51.8%)	116 (49.2%)	279 (53.0%)	
Inner regional	196 (25.7%)	56 (23.7%)	140 (26.6%)	0.016*
Outer regional	150 (19.7%)	61 (25.8%)	89 (16.9%)	
Remote or very remote	21 (2.8%)	3 (1.3%)	18 (3.4%)	
**Self-rated health**				
**Physical health**				
Excellent	24 (3.0%)	13 (5.2%)	11 (2.0%)	
Very good	146 (18.0%)	42 (16.7%)	104 (18.6%)	0.067
Good	280 (34.5%)	95 (37.7%)	185 (33.1%)	
Fair	267 (32.9%)	76 (30.2%)	191 (34.2%)	
Poor	94 (11.6%)	26 (10.3%)	68 (12.2%)	
**Mental health**				
Excellent	74 (9.2%)	31 (12.4%)	43 (7.7%)	
Very good	182 (22.5%)	66 (26.5%)	116 (20.8%)	0.037*
Good	213 (26.4%)	61 (24.5%)	152 (27.2%)	
Fair	236 (29.2%)	66 (26.5%)	170 (30.4%)	
Poor	103 (12.7%)	25 (10.0%)	78 (14.0%)	
**No. GP visits past 12 months**	Mean = 6.9 (SD = 6.2)	6.7 (6.0%)	7.0 (6.2%)	0.470

TAFE = Technical and Further Education.

**Table 2.** Characteristics of smokers

Types of tobacco smoked	Cigarettes only	N = 537 (87.9%)
	Cigarettes and other products	N = 49 (8.0%)
	Only other types	N = 19 (3.1%)
Kind of cigarettes	Only factory made	N = 381 (66.0%)
	Factory made and own rolled	N = 122 (21.1%)
	Own rolled	N = 74 (12.8%)
Vaping or use e-cigarettes	Daily use	N = 16 (2.7%)
	Occasional use	N = 35 (8.6%)
	Past use (not current)	N = 141 (23.7%)
	Never used	N = 403 (67.7%)
Time from waking to first cigarette	<30 minutes	N = 453 (83.1%)
	>30 minutes	N = 92 (16.9%)
Difficulty refraining from smoking in prohibited places	Yes	155 (28.5)
	No	388 (71.5)
Cigarettes smoked/day	Median = 20.0 (IQR = 10.0)	
Smoke even if so ill you are in bed most of the day	Yes	N = 316 (58.1%)
	No	N = 228 (41.9%)
How important is it for you to quit smoking?	Not important	N = 19 (3.4)
	Slightly/moderately important	N = 45 (8.1%)
	Important/very important	N = 476 (85.2%)
In the past 12 months, were you advised to quit smoking by a GP or other care provider at your GP practice?	Never	N = 111 (19.6%)
	Sometimes	N = 178 (31.5%)
	Usually	N = 130 (23.0%)
	Always	N = 146 (25.8%)
Did you try to quit smoking?	No	N = 160 (35.9%)
	Yes	N = 286 (64.1%)
Last quit attempt	<12 months ago	N = 290 (51.4%)
	>12 months ago	N = 230 (40.9%)
	Never	N = 44 (7.8%)

Most smokers (85.2%) indicated that it was ‘very important’ or ‘important’ to them to quit smoking and nearly two in three (64.1%) reported they had tried to quit smoking, with more than half (51.4%) having tried to quit smoking within the last 12 months. Despite this, fewer than half (48.8%) recalled being advised to quit smoking (‘usually’ or ‘always’) by their GPs in the last 12 months.

### Differences in patient experience between smokers and ex-smokers

Relational experiences of care such as feeling listened to, being shown respect, and having felt GPs spent enough time with them did not differ between smokers and ex-smokers (Table 3). However, current smokers were significantly more likely to delay seeking care or avoid going to a GP when needed (Table 3). Anticipated stigma was significantly higher among current smokers compared to ex-smokers (mean = 10.2 vs 8.4, p < 0.001) and tended to decline the longer a person had quit smoking (Rho = -0.243, p < 0.001). Overall rating of care did not differ between smokers and ex-smokers (mean = 8.0 vs 7.9, p = 0.848).

**Table 3.** Differences between smokers^ and ex-smokers in experience of care

	Smoking status		Test statistic	95% CI for EXP(B)*
	Ex-smoker	Current smoker		
**GPs listened carefully**			Exp(B)	
Sometimes/rarely/never	N = 57 (21.7%)	N = 144 (25.9%)	Ref	
Often	N = 70 (26.6%)	N = 133 (23.9%)	0.774	0.486–1.233
Always	N = 136 (51.7%)	N = 280 (50.3%)	0.789	0.520–1.199
**GPs showed respect**			Exp(B)	
Sometimes/rarely/never	N = 60 (22.9%)	N = 128 (22.9%)	Ref	
Often	N = 62 (23.7%)	N = 127 (22.7%)	1.017	0.633–1.633
Always	N = 140 (53.4%)	N=304 (54.4%)	0.996	0.662–1.499
**GPs spent enough time**			Exp(B)	
Sometimes/rarely/never	N = 71 (27.1%)	N =164 (29.3%)	Ref	
Often	N = 59 (22.5%)	N = 127 (22.7%)	1.059	0.671–1.671
Always	N = 132 (50.4%)	N = 268 (48.7%)	0.909	0.616–1.343
**Clerks and receptionists were helpful**			Exp(B)	
Sometimes/rarely/never	N = 49 (18.8%)	N = 121 (22.0%)	Ref	
Often	N = 80 (30.8%)	N = 168 (30.5%)	1.119	0.770–1.627
Always	N = 131 (50.4%)	N = 261 (47.5%)	1.318	0.855–2.033
**Clerks and receptionists treated you with courtesy and respect**			Exp(B)	
Sometimes/rarely/never	N = 29 (11.1%)	N = 74 (13.4%)	Ref	
Often	N = 59 (22.6%)	N = 131 (23.8%)	1.083	0.735–1.597
Always	N = 173 (66.3%)	N = 346 (62.8%)	1.182	0.713–1.960
**Needed to go to GP but DIDN’T go**			Exp(B)	
Did not go to GP	N = 109 (41.8%)	N = 303 (53.5%)	**1.544**	**1.096-2.175**
Went to GP when needed	N = 152 (58.2%)	N = 263 (46.5%)		
**Needed to go to GP but DELAYED going**			Exp(B)	
Delayed going to GP	N = 147 (55.9%)	N = 371 (65.9%)	**1.544**	**1.097–2.174**
Went to GP when needed	N = 116 (44.1%)	N = 192 (34.1%)		
**Got appointment as soon as needed**			Exp(B)	
Sometimes/never	N = 49 (19.4%)	N = 127 (24.4%)	0.752	0.496–1.139
Always/usually	N = 203 (80.6%)	N = 394 (75.6%)		
**Anticipated stigma Total score**	Mean = 8.4/20 (SD = 3.8)	Mean =10.2/20 (SD = 3.8)	Beta = 0.206	**1.119–2.288**
	Time since quit^ <12 months–8.8 (3.6) 1–2 years–8.6 (4.3) 3–5 years–7.4 (3.7)			
**Overall rating of GPs** (10 is highest)	Mean = 8.0/10 (SD = 2.1)	Mean = 7.9/10 (SD = 2.2)	Beta = -0.007	-3.263

*Adjusted for age, sex, Socio-Economic Index For Area (SEIFA) decile, education, remoteness, and self-rated health; ^Excludes current smokers who only used ‘other tobacco’ products.

### Patient experience and frequency of smoking cessation advice

Respondents who reported their GP or other healthcare provider at their GPs’ practices advised them to quit smoking ‘always or usually’ were most likely to report their GPs ‘always’ listened carefully, showed respect, and spent enough time with them (Table 4) and advised them in a way that motivated them to try to quit smoking (Table 4). Overall rating of GPs’ care was higher when respondents reported their GP always or usually advised them to quit smoking and advised them in a way that motivated them to try to quit (Table 4).

**Table 4.** Logistic regression analyses of patient experience of care by advice to quit smoking

*Current Smokers*	*How often were you advised to Quit smoking by a GP*			*Did they advise you in a way that motivated you to try to quit*		
	*Sometimes/never*	*Always/usually*	*OR (95% CI)*	*No/not really*	*Yes*	*OR (95% CI)*
	*(n = 289, 51.2%)*	*(n = 276, 48.8%)*		*(n = 258, 57%)*	*(n = 195, 43%)*	
**GPs listened carefully**						
Sometimes/rarely/never	N = 92 (31.8%)	N = 59 (21.5%)	Ref	N = 99 (38.7%)	N = 10 (5.1%)	Ref
Often	N = 62 (21.5%)	N = 71 (25.9%)	**1.92 (1.15–3.21)**	N = 69 (27.0%)	N = 45 (23.1%)	**7.29 (3.25–16.32)**
Always	N = 135 (46.7%)	N = 144 (52.6%)	**1.73 (1.11–2.70)**	N = 88 (34.4%)	N = 140 (71.8%)	**17.90 (8.31–38.56)**
**GPs showed respect**						
Sometimes/rarely/never	N = 78 (27.0%)	N = 54 (19.6%)	Ref	N = 86 (33.3%)	N = 12 (6.2%)	Ref
Often	N = 63 (21.8%)	N = 63 (22.8%)	1.61 (0.95–2.74)	N = 65 (25.2%)	N = 37 (19.0%)	**3.64 (1.72–7.69)**
Always	N = 148 (51.2%)	N = 159 (57.6%)	**1.77 (1.13–2.79)**	N = 107 (41.5%)	N = 146 (74.9%)	**9.74 (4.90–19.34)**
**GPs spent enough time**						
Sometimes/rarely/never	N = 94 (32.5%)	N = 73 (26.4%)	Ref	N = 108 (41.9%)	N = 22 (11.3%)	Ref
Often	N = 67 (23.2%)	N = 60 (21.7%)	1.12 (0.68–1.84)	N = 59 (22.9%)	N = 40 (20.5%)	**3.45 (1.81–6.58)**
Always	N = 128 (44.3%)	N = 143 (51.8%)	**1.60 (1.04–2.46)**	N = 91 (35.3%)	N = 133 (68.2%)	**7.11 4.03–12.57)**
**Needed to go to GP**						
**but DIDN’T go**						
Didn’t go to GP	N = 146 (50.5%)	N = 156 (56.7%)		N = 152 (58.9%)	N = 98 (50.5%)	
Went to GP when needed	N = 143 (49.5%)	N = 119 (43.3%)	0.86 (0.60–1.25)	N = 106 (41.1%)	N = 96 (49.5%)	1.44 (0.96-2.17)
**Needed to go to GP**						
**but DELAYED going**						
Delayed going to GP	N = 173 (60.3%)	N = 197 (71.6%)		N = 183 (70.9%)	N = 127 (65.5%)	
Went to GP when needed	N = 114 (39.7%)	N = 78 (28.4%)	**0.67 (0.45–0.99)**	N = 75 (29.1%)	N = 67 (34.5%)	1.32 (0.85–1.76)
**Got appointment as soon as needed**						
Sometimes/never	70 (26.4%)	55 (21.8%)		65 (27.5%)	33 (18.2%)	
Always/usually	195 (73.6%)	197 (78.2%)	1.19 (0.77–1.84)	171 (72.5%)	148 (81.8%)	1.63 (0.98–2.70)
**Anticipated stigma**	9.6/20 (3.8)	10.8/20 (3.7)	**1.07 (1.02–1.13)**	11.0 (4.0)	9.4 (3.3)	**0.89 (0.84–0.94)**
**Overall rating of GPs (/10)**	7.7/10 (2.3)	8.1/10 (2.0)	**1.13 (1.03–1.23)**	7.3 (2.2)	8.9 (1.4)	**1.67 (1.45–1.93)**

*Adjusted for age, gender, Socio-Economic Index For Area (SEIFA) decile, education, remoteness, and self-rated health.

Conversely, smokers reporting more frequent advice to quit smoking also reported greater anticipation of stigma in health settings (10.8 vs 9.6, p < 0.001) (Table 4), and respondents who always or usually were advised to quit smoking were 33% less likely to go to their GP when they needed (Table 4).

## Discussion

The principal finding from this study is that asking patients who smoke about their smoking behaviour did not negatively impact the rating of the care that they received from GPs. It seems that patients realize that GPs have an important role in asking about smoking and this is likely seen as an indicator of high-quality care. Despite this, up to one-third of respondents seemed to dread these questions and reported delaying or avoiding seeking health care when they needed it. Patients who reported they were advised to quit smoking in a way that motivated them to try to quit felt listened to and felt their GPs showed respect and spent enough time with them. Relational experiences of care (feeling listened to, being shown respect, having enough time with GPs) were rated the same between patients who were current smokers and those who were ex-smokers. Of concern, however, was the finding that respondents who were current smokers reported higher scores for anticipation of stigma and were most likely to delay or avoid going to a GP when needed.

Our finding that the overall rating of care received from GPs was perceived to be higher among those who were more frequently advised to quit smoking is consistent with patient experience surveys conducted in the USA (Winpenny *et al.*, 2017, Holla *et al.*, 2018), as well as other studies of smokers satisfaction with primary care (Conroy *et al.*, 2005, Barzilai *et al.*, 2001). Together, these findings provide support for recommendations in smoking cessation guidelines that healthcare providers should provide advice to quit smoking ‘at every opportunity’ and that healthcare providers can approach this task with confidence. The combination of higher overall ratings and more positive ratings of relational experiences of care neutralizes concerns that advising smokers to quit smoking may damage the doctor–patient relationship (Coleman *et al.*, 2000).

However, these messages need to be provided sensitively. One concerning finding was that ratings of patient experience among this sample were substantially lower than those reported for the general primary care population in Australia. In particular, the higher anticipation of stigma in health settings reported by this group was a concern, particularly among the group who were current smokers.

‘Anticipated’ stigma reflects the extent to which people expect to experience stereotyping, prejudice, and discrimination directed at them from others in the future (Earnshaw and Quinn, 2011, Earnshaw *et al.*, 2012). It can arise from internalized stigma and experiences of stigma but also from fear of discrimination or stereotyping and judgement in the future and is an important predictor of delayed and avoided help-seeking among primary care patients with chronic respiratory illness (Madawala *et al.*, 2023a). The concept encompasses the extent to which people think a status is stigmatized in their environment and not just what they personally have experienced (Earnshaw *et al.*, 2013).

These results are consistent with studies of other stigmatized populations in primary care. The language healthcare providers use in speaking with these patients can be different and unconscious or unintentional bias can result in people being treated differently or unfairly and contribute to experiences of stigma in healthcare settings (Wilson, 2020, Thille, 2019) and loss of trust (Kasiviswanathan *et al.*, 2025). Our findings that anticipated stigma is lower once patients quit smoking and tends to slowly decline over time but can persist up to five years from when an individual has quit smoking.

## Limitations

This study aimed to reach a general population aged 35 years and over, responding to advertisements on Facebook and Instagram. It is possible that users of these services with more negative experiences may have been more likely to complete the survey, possibly explaining the overall more negative views expressed here about experience of care, compared with representative samples. As such further studies, utilizing representative sampling techniques, are required to confirm the findings presented here. Data collection for the study was completed just prior to the COVID-19 pandemic, and so the findings do not reflect any changes in healthcare access and experience in primary care during the pandemic.

Using social media provided an opportunity to recruit a sample from across Australia. Social media has been found to be a viable recruitment method for current and ex-smokers, who can be challenging to reach and recruit into research if they have experienced smoking-related stigma (Carter-Harris *et al.*, 2016, Frandsen *et al.*, 2014). We limited participation to individuals aged 35 years and older to allow for a greater range of smoking-related experiences and risk for smoking-related illness. The results should not be generalized to younger smokers and ex-smokers who may have different experiences of care to older adults. Nonetheless, the characteristics of smokers in our sample closely reflect characteristics of smokers reported in Australia’s 2019 National Drug Strategy Household Survey in terms of types of cigarettes smoked, use of e-cigarettes, and the proportion who had tried to quit but were not successful, although our sample tended to smoke more cigarettes per day than the national average.

A further limitation relates to our assessment of patient experience of care. We measured experience by self-report, using items from the Australian Bureau of Statistics (ABS) Patient Experience Survey. These questions asked respondents about all experiences of care (in general practice) but not about specific episodes of care. As such, our results need to be interpreted as general perceptions of encounters with primary care providers, not specific ones in which smoking was mentioned (if it was in some). We did not ask about the number of different GPs participants had visited or if care was provided by a preferred or regular GP. Ratings of care may be different if a single point of reference was used or if questions were asked about a preferred or usual provider. Lastly, these questions ask about experiences of care in the past 12 months, and there is a risk of recall bias, particularly if care had not been sought recently.

## Conclusion

Frequent advice to quit smoking appears acceptable to primary care patients with a smoking history, especially from GPs who provide advice in a way that is motivating to the smoker. This study provides further strength to evidence that the provision of smoking advice at every visit, as recommended by guidelines, does not negatively impact the doctor–patient relationship or ratings of care. In fact, higher ratings of care were given by patients who were asked about smoking cessation more frequently, an outcome that has been linked to better quality of care in general (Winpenny *et al.*, 2017). However, this needs to be balanced with the concerning finding of delayed help seeking, particularly for patients who expect their GP to ask them about their smoking. This emphasizes the importance of asking about smoking and providing advice to quit in a supportive, non-judgemental way, by partnering with the patient, sharing power, and listening to them carefully.

## Data Availability

The data underlying this article will be shared on reasonable request to the corresponding author.
